# A Potential Intracanal Medicament, 2-Hydroxyisocaproic Acid (HICA): Cytotoxicity, Genotoxicity, and Its Effect on SCAP Differentiation

**DOI:** 10.3390/dj11120270

**Published:** 2023-11-27

**Authors:** Cristine Smoczer, Yun K. Park, James B. Herrington, Mazin A. Askar, Sarah Plecha, Eric Krukonis, Susan B. Paurazas

**Affiliations:** 1 Division of Integrated Biomedical Sciences, University of Detroit Mercy School of Dentistry, Detroit, MI 48208, USA; smoczecr@udmercy.edu (C.S.); splecha@hfcc.edu (S.P.); krukones@udmercy.edu (E.K.); 2 Graduate Endodontics, University of Detroit Mercy School of Dentistry, Detroit, MI 48208, USAaskarma@udmercy.edu (M.A.A.)

**Keywords:** 2-hydroxyisocaproic acid, SCAP, cytotoxicity, genotoxicity, odonto/osteogenic differentiation, regenerative endodontic procedures

## Abstract

Intracanal medicaments with maximal antimicrobial efficacy and minimal damage to resident stem cells are essential for successful regenerative endodontic procedures. 2-Hydroxyisocaproic acid (HICA) could have the attributes of a potential intracanal medicament. This study evaluates its cytotoxicity, genotoxicity, and effects on the odontogenic and osteogenic differentiation of the stem cells of the apical papilla (SCAP). Cytotoxicity and cell viability assays were performed on cells treated for 24, 48, and 72 h with varying concentrations of HICA and compared to the standard intracanal medicament, calcium hydroxide. The genotoxicity was assessed via immunofluorescence for two markers of DNA double-strand breaks: phosphorylated γH_2_AX and 53BP1. The SCAP differentiation was evaluated based on the alkaline phosphatase activity, Alizarin Red staining, and expression of odontogenic and osteogenic genes (*DSPP1*, *BSP1*, *OCN*, *RUNX2*) in the presence of selected HICA concentrations. HICA was not cytotoxic at concentrations up to 10 mg/mL, regardless of the exposure time, although it was cytostatic at all tested concentrations. HICA was not genotoxic at concentrations below 5 mg/mL. No difference in cytotoxicity or genotoxicity was found between HICA and calcium hydroxide at 1 mg/mL. HICA retained about 70% of the osteogenic differentiation potential at 1 mg/mL. Within the limitations of this in vitro study, we show that HICA at 1 mg/mL could be a potential intracanal medicament for REPs.

## 1. Introduction

Regenerative endodontic procedures (REPs) have emerged as a viable treatment option for necrotic pulps in immature teeth [1]. Given the small diameter of the apex and the weak dentinal walls in immature teeth, the expert consensus in approaching REPs is that mechanical instrumentation should be replaced by chemical debridement of the canal space [2,3]. Therefore, it is imperative to use disinfection irrigants and intracanal medicaments with maximum antibacterial efficacy and minimum cytotoxicity to the resident stem cells [4]. Stem cells of the apical papilla (SCAP) in immature permanent teeth are the main stem cells that enter the canal system when bleeding is evoked during REPs [5]. They are mesenchymal stem cells with demonstrated osteogenic and odontogenic differentiation potential, which needs to be maintained for the successful regeneration of a functional pulp–dentin complex [6,7].

The American Association of Endodontics (AAE) guidelines (2021) recommend calcium hydroxide, triple antibiotic paste (TAP), modified TAP (mTAP), and double antibiotic paste (DAP) as intracanal medicaments to act on the bacteria in the root canal system [3]. However, these substances vary in their antimicrobial activity and their effects on stem cells. Although highly efficacious antibacterials, the cytotoxic effect of TAPs, mTAP, and DAP on SCAP ranged in a dose-dependent fashion, from no impact to total loss of cell viability [8,9,10,11,12]. Calcium hydroxide, a widely used intracanal medicament has similar disinfection ability [13] and lower cytotoxic potential than the antibiotic pastes [9]. Some studies showed significant increases in SCAP proliferation, while others showed no change in cytotoxicity depending on the concentration [8,9,10]. Propolis, a natural resin produced by honeybees, was shown to be significantly less cytotoxic than calcium hydroxide [14], while exhibiting antibacterial efficacy similar to that of TAP and calcium hydroxide [15,16,17], with additional antiviral, anti-inflammatory, and antioxidant properties [18]. Although propolis has good biocompatibility and antimicrobial effects, which makes it a good candidate for a natural intracanal medicament, it should be used with caution *in vivo* because of its allergenic potential [19].

Given the heterogeneity in the reported properties of common intracanal medicaments [20], the search for a biocompatible intracanal medicament with good antimicrobial properties is ongoing. 2-Hydroxyisocaproic acid (HICA) is a natural metabolite in the leucine degradation pathway in humans and certain *Lactobaccilus* species. In humans, it is produced in the muscles and connective tissue and detected in low concentrations in plasma [21]. HICA is also a microbial metabolite produced mainly by lactic acid bacteria in the process of animal protein fermentation [22]. HICA was shown to have broad-spectrum antimicrobial activity against most Gram-positive and Gram-negative species and broad antifungal activity against *Candida* and *Aspergillus* species [23,24,25,26,27,28]. It also displayed superior antimicrobial activity against *Entercoccus faecalis* and provided enhanced disruption of the biofilm and better penetration of dentine tubules in *ex vivo* teeth when compared to calcium hydroxide [24,25,27]. Additionally, HICA’s minimal inhibitory concentration against some relevant dental bacterial species was 1 mg/mL, as compared to 16 mg/mL for calcium hydroxide [29]. HICA was also found to be less cytotoxic and genotoxic to human periodontal ligament fibroblasts than calcium hydroxide at similar concentrations [30]. This study evaluates the cytotoxicity and genotoxicity of HICA and its effect on SCAP differentiation to odontogenic and osteogenic lineages.

## 2. Materials and Methods

### 2.1. Cells and Reagents

The SCAP RP-89 cells [31] were provided by Dr. Diogenes and used in subsequent experiments at passages 5–8. Cells cultured in α-minimum essential medium (Gibco, Grand Island, NY, USA) supplemented with 10% fetal bovine serum (Gemini, West Sacramento, CA, USA), 1% L-glutamine, and 1% penicillin/streptomycin (Sigma-Aldrich, St. Louis, MO, USA) were grown at 37 °C and in 5% CO_2_.

The DL-2 HICA (Sigma-Aldrich, St. Louis, MO, USA) was prepared as a 40 mg/mL stock solution in cell culture medium and neutralized to pH 7.0. The stock solution was diluted 10, 5.0, 2.5, and 1.0 mg/mL in cell culture medium for use in subsequent experiments. The calcium hydroxide (Sigma-Aldrich, St. Louis, MO, USA) was prepared by diluting a 10 mg/mL stock solution to 1 mg/mL in culture medium.

### 2.2. Kinetic Cytotoxicity Assay

SCAP seeded at 10,000 cells/well in a black 96-well plate were allowed to adhere for 24 h before treatment with varying dilutions of HICA and Ca(OH)_2_ in cell culture media. The kinetic cytotoxicity levels at 24, 48, and 72 h post-treatment were evaluated by using the CellTox Green Assay (Promega, Madison, WI, USA), with the relative fluorescence intensity of the samples read on a Tecan Spark plate reader. The negative control was represented by cells in cell culture medium without any treatment. The background fluorescence of the cell culture medium alone was subtracted from the fluorescence readings of each treatment group.

### 2.3. Cell Viability Assay

The SCAP were seeded at 15,000 cells/well in a 96-well black opaque-walled plate and allowed to adhere for 24 h before treatment with varying concentrations of HICA and Ca(OH)_2_. The cell viability at 72 h of treatment was assessed by using the CellTiter-Glo Assay (Promega, Madison, WI, USA), as per the manufacturer’s protocol. Cells in media only were used as a negative control. The background luminescence was established in wells with media without cells. The relative luminescence units (RLUs) were calculated by subtracting the background luminescence from the luminescence readings of each group. The no treatment control was set as 100% cell viability and the SCAP viability in all treatment groups was calculated relative to the negative control.

### 2.4. Immunofluorescence

The DNA double-strand breaks produced after treatment of SCAP with varying concentrations of HICA and Ca(OH)_2_ were examined by immunofluorescence. The SCAP were seeded at 40,000 cells/well in a 12-well plate containing glass coverslips (#12-545-80; Fisher Scientific, Waltham, MA, USA) and allowed to adhere for 24 h before treatments. Untreated cells were used as a negative control, and cells treated with hydrogen peroxide at 1 mmol/L for 10 min were used as a positive control. Following 24 h of treatment with different concentrations of HICA or Ca(OH)_2_, the cells were fixed in 4% paraformaldehyde (pH = 7.4) for 10 min. After fixation, the cells were permeabilized with 0.1% Triton X-100 (151-0407; Bio-Rad, Hercules, CA, USA) in PBS for 15 min and blocked with 3% bovine serum albumin (A9647; Sigma-Aldrich, St. Louis, MO, USA) in PBS for 30 min. The coverslips were then incubated with primary antibodies at a dilution of 1:400 for anti-phospho-γH2AX Ser139 (05-636-I; Millipore Sigma, Burlington, MA, USA) and 1:500 for anti-53BP1 (NB100-304; Novus Biologicals, Centennial, CO, USA) overnight at 4 °C, followed by incubation at room temperature for 1.5 h with the combined secondary antibodies goat anti-mouse Alexa Fluor488 (A-11001, Thermo Fisher Scientific, Waltham, MA, USA) and goat anti-rabbit Alexa Fluor568 (A-11011, Thermo Fisher Scientific, Waltham, MA, USA) at 1:250 dilution. The coverslips were mounted onto slides using ProLong Gold Antifade Mountant with DAPI (P36941, Thermo Fisher Scientific, Waltham, MA, USA).

Images were acquired using a fluorescence microscope at 40× magnification (BX63; Olympus, Tokyo, Japan). The cells were counted by a blinded investigator and cells with 4 or more foci of colocalized gH2AX and 53BP1 were considered to have DNA damage. The number of cells positive for genotoxicity in a total of 100 counted cells was determined for each condition.

### 2.5. Cell Culture for Differentiation

To induce osteogenic differentiation, SCAP were seeded at 50,000 cells/well in 6-well plates in osteogenic differentiation media (ODM) obtained via the addition of 10 mM of β-glycerophosphate, 0.2 mM of L-ascorbic acid, and 0.1 μM of dexamethasone (all from Sigma-Aldrich, St. Louis, MO, USA) to complete the α-MEM cell culture media. The SCAP were treated with varying dilutions of HICA in ODM. Cells grown in ODM without any treatments were used as a positive control, while cells grown in regular medium were used as a negative control. The media were changed every 3–4 days for the duration of the study.

### 2.6. Alkaline Phosphatase Activity

The alkaline phosphatase (ALP) activity was evaluated by using the Alkaline Phosphatase Activity Assay (#8285, ScienCell, Carlsbad, CA, USA) in SCAP grown in media with and without HICA and harvested at day 14. The cells were processed per the manufacturer’s protocol and the AP activity was determined based on the absorbance at 405 nm.

### 2.7. Alizarin Red Staining

The SCAP were cultured for 15 days in ODM with HICA and in ODM without HICA for an additional 15 days. After 30 days, the SCAP mineralization was evaluated by using the Alizarin Red S Staining Quantification Assay (#8678, ScienCell, Carlsbad, CA, USA) per the manufacturer’s protocol. The Alizarin Red-stained cells were imaged prior to quantitation of the eluted staining based on the absorbance at 570 nm.

### 2.8. Gene Expression of Osteogenic Markers

SCAP grown in ODM with and without HICA for 7 days were harvested and the total RNA was extracted using Tri-Reagent (#AM9738, Invitrogen, Waltham, MA, USA). The first-strand cDNA was synthesized from 200 ng of RNA using the iScript cDNA synthesis kit (#1708891, BioRad, Hercules, CA, USA). PCR amplification for *DSPP1, BSP1, OCN,* and *RUNX2* was performed on a BioRad CFX96 Real-Time Detection System using the iQ SYBR Green Supermix (#1708880, BioRad, Hercules, CA, USA) with 1 μg of cDNA and the corresponding primer pair shown in Table 1. The thermal cycling settings included denaturation at 95 °C for 3 min followed by 40 cycles of 10 s at 95 °C, 30 s at 56 °C, and 30 s at 72 °C. The gene expression was normalized to *GAPDH* by using the 2^−δδCt^ method.

### 2.9. Statistical Analysis

The experiments were performed in triplicate and the results are presented as means ± the standard deviation. The data were analyzed using a one-way ANOVA followed by a post hoc Tukey’s test for most experiments. The kinetic cytotoxicity data were analyzed using a two-way ANOVA followed by a post hoc Tukey’s test. The statistical significance was set at *p* < 0.05. The data analysis was performed using GraphPad Prism 9.4 software (GraphPad Software, Boston, MA, USA).

## 3. Results

### 3.1. HICA Biocompatibility to SCAP

**Figure 1:**
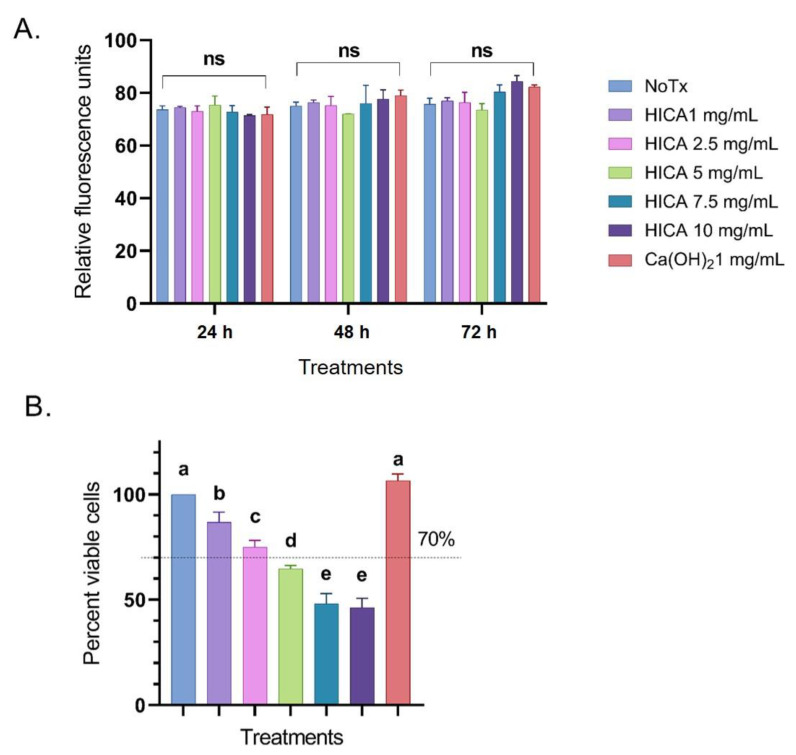
Effects of HICA and calcium hydroxide on SCAP survival. (**A**) Cytotoxicity levels of HICA at concentrations of 1–10 mg/mL and 1 mg/mL of calcium hydroxide were evaluated after SCAP exposure for 24, 48, and 72 h and represented as relative fluorescence units. (**B**) Viability levels of SCAP exposed to varying concentrations of HICA and calcium hydroxide for 72 h exposure are presented as percentages relative to the no treatment control set to 100%. Data are presented as the means ± standard deviation of the mean. Cytotoxicity data were analyzed using a two-way ANOVA followed by post hoc Tukey’s test, while viability data were analyzed using a one-way ANOVA followed by post hoc Tukey’s test. Different letters (a, b, c, d, e) indicate statistically significant differences between treatment groups (*p* < 0.05); the same letter indicates no statistically significant difference between treatments (*p* > 0.05); ns: not significant (*p* > 0.05).

We evaluated the cytotoxicity levels of HICA concentrations varying from 1 to 10 mg/mL and compared them to 1 mg/mL of Ca(OH)_2_ by using the CellTox Green Cytotoxicity Assay after 24, 48, and 72 h of treatment exposure. There was no significant difference in cytotoxicity for any HICA concentrations compared to the control (*p* > 0.05). The maximum cytotoxicity for all evaluated treatments was reached after 24 h of exposure and prolonged exposure did not have a significant impact (Figure 1A).

The SCAP viability after exposure to varying concentration of HICA and Ca(OH)_2_ for 72 h was examined using the CellTiter Glo Assay, with the untreated control set to 100%. HICA exposure significantly reduced the cell viability at all tested concentrations in a dose-dependent manner, with concentrations above 2.5 mg/mL decreasing the cell viability to below 70% (*p* < 0.05). Calcium hydroxide caused no reduction in cell viability at 1 mg/mL (*p* > 0.05) (Figure 1B).

### 3.2. HICA Genotoxicity to SCAP

**Figure 2:**
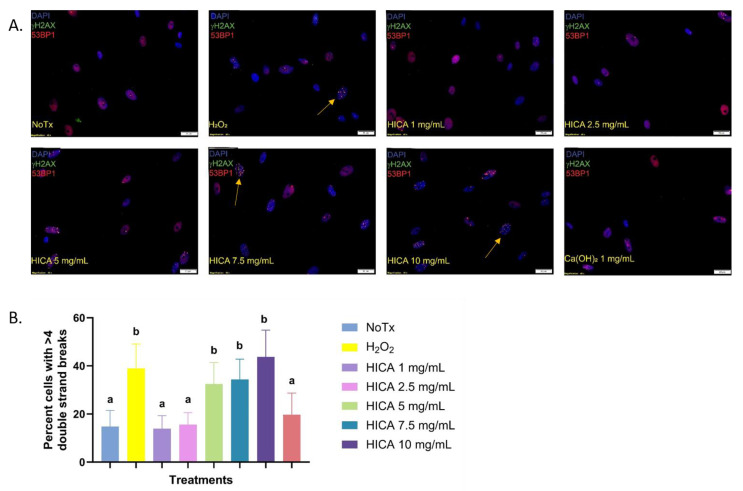
HICA genotoxicity evaluated based on immunofluorescence for two markers of DNA double-strand breaks: γH2AX and 53BP1. (**A**) Representative fluorescence microscopy images of SCAP exposed to selected concentrations of HICA. Nuclei were stained with DAPI and imaged in the blue channel, while phosphorylated γH2AX foci are imaged in the green channel and 53BP1 foci in the red channel. (**B**) The percentages of cells displaying more than 4 co-localized γH2AX/53BP1 foci (indicated by arrows) after treatment with varying HICA concentrations. Hydrogen peroxide served as the positive control for the induction of double-strand breaks. Data are expressed as the means ± standard deviation from 3 independent experiments and were analyzed using a one-way ANOVA followed by post hoc Tukey’s test. Different letters (a, b) indicate statistically significant differences between treatment groups (*p* < 0.05); the same letter indicates no statistically significant difference between treatments (*p* > 0.05).

The potential genotoxic effect of HICA and calcium hydroxide was evaluated based on the number of induced DNA double-strand breaks (DBS) after exposure to these substances for 24 h. The co-localization foci of phosphorylated γH2AX and 53BP1 were considered to account for real DBS (Figure 2A). Cells exhibiting more than four co-localization foci were counted and their percentage from the total number of cells was calculated. Here, 1 and 2.5 mg/mL of HICA and 1 mg/mL of Ca(OH)_2_ displayed no genotoxicity differing from the untreated cells, while HICA concentrations above 2.5 mg/mL showed genotoxicity levels similar to those of a known genotoxic agent, hydrogen peroxide (Figure 2B).

### 3.3. HICA Effect on SCAP Differentiation

To induce osteogenic or odontogenic differentiation, SCAP were grown in osteogenic differentiation media (ODM) with and without 1 mg/mL of HICA, and the activity and gene expression levels of various differentiation markers were evaluated at 7, 14, and 30 days.

**Figure 3:**
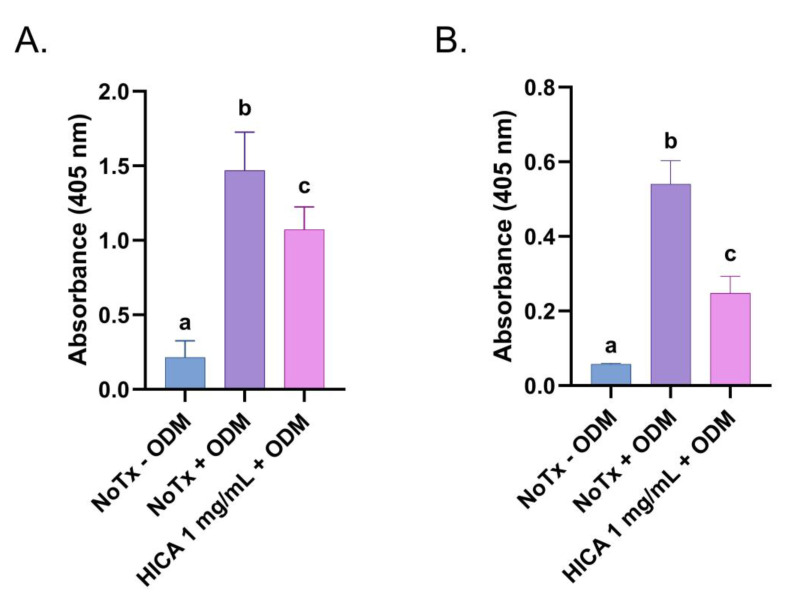
Effects of HICA on odontogenic and osteogenic differentiation of SCAP. (**A**) Alkaline phosphatase activity in SCAP exposed to regular medium, osteogenic differentiation media (ODM) without any treatment, or with the addition of 1 mg/mL of HICA for 14 days. (**B**) Quantitative detection of Alizarin Red released from stained mineralization nodules in SCAP allowed to differentiate for 30 days with or without addition of 1 mg/mL of HICA. Data are expressed as means ± the standard deviation from 3 independent experiments and were analyzed using a one-way ANOVA followed by post hoc Tukey’s test. Different letters (a, b, c) indicate statistically significant differences between treatment groups (*p* < 0.05); the same letter indicates no statistically significant difference between treatments (*p* > 0.05).

We evaluated the enzymatic activity of alkaline phosphatase, a ubiquitous marker of osteoblastic differentiation, after 14 days of SCAP exposure to ODM with and without HICA. Treatment with 1 mg/mL of HICA resulted in a 37% reduction in ALP activity compared to the no treatment group (Figure 3A). We observed similar results for the quantitative and qualitative detection of mineralization nodules via Alizarin Red staining after 30 days incubation. At 1 mg/mL, HICA retained about 46% of the mineralization potential (Figure 3B).

**Figure 4:**
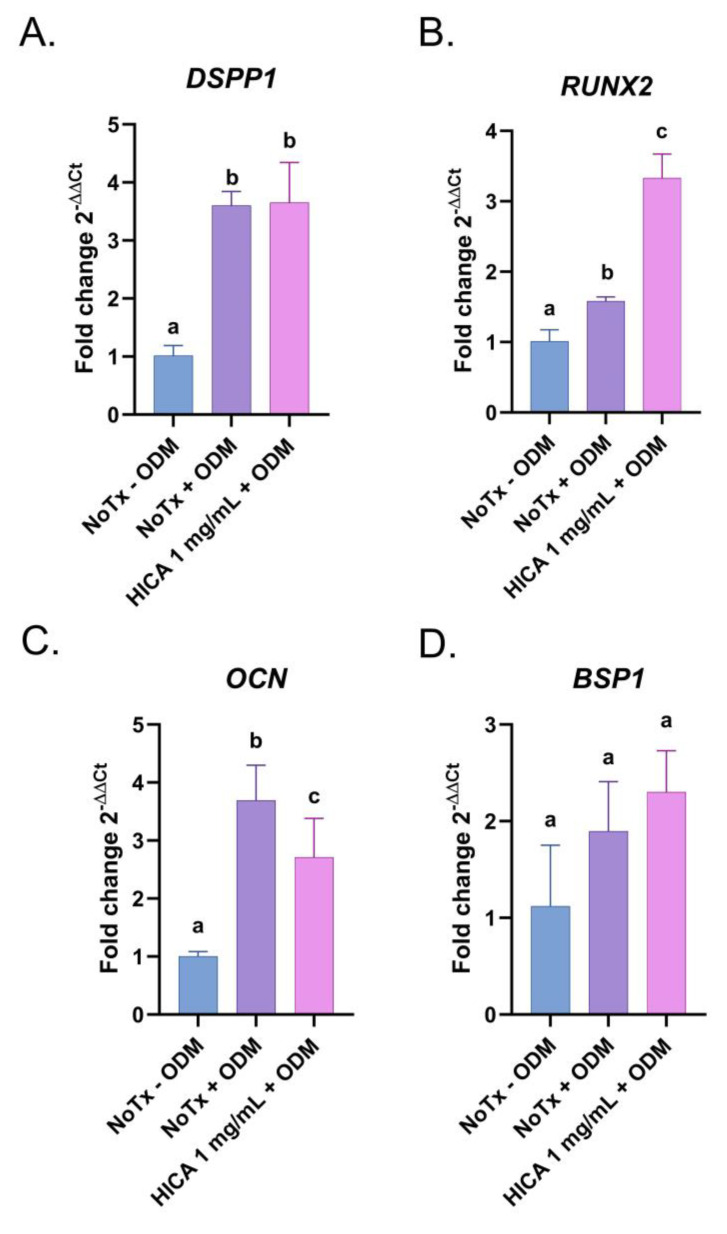
Expression of odontogenic and osteogenic markers in SCAP treated with HICA. SCAP were exposed for 7 days to osteogenic differentiation media with and without 1 mg/mL of HICA and the expression of each gene was determined via RT-PCR and normalized to *GAPDH*. Fold changes in gene expression of (**A**) *DSPP1*, (**B**) *RUNX2*, (**C**) *OCN*, and (**D**) *BSP1* were determined by comparing them to the normal cell culture medium control. Data are representative of 3 independent experiments and were analyzed using a one-way ANOVA followed by post hoc Tukey’s test. Different letters (a, b, c) indicate statistically significant differences between treatment groups (*p* < 0.05); the same letter indicates no statistically significant difference between treatments (*p* > 0.05).

We further examined the gene expression of known osteogenic and odontogenic markers, such as *dentin sialoprotein 1 (DSPP1)*, *bone sialoprotein 1 (BSP1)*, *osteocalcin (OCN)*, and *runt-related transcription factor 2 (RUNX2)*, in SCAP exposed to ODM with and without HICA for 7 days. The expression levels of *DSPP1*, a marker of mature odontoblasts, and *BSP1*, the initiator of crystal nucleation, were similar in the osteogenic media with and without the addition of 1 mg/mL of HICA (Figure 4A,D). *RUNX2,* the marker for the induction of osteoblast differentiation, was induced in HICA-treated groups (Figure 4B). *OCN*, a late downstream target of *RUNX2,* showed a slight decrease from no treatment when exposed to 1 mg/mL of HICA (Figure 4C).

## 4. Discussion

Effective root canal disinfection is critical in achieving the resolution of the signs and symptoms of infection, which is the primary objective of REPs. Due to the thin dentinal walls of immature teeth, minimal to no instrumentation is advised in REPs according to the 2021 AAE Regenerative Treatment Protocol [3]. The disinfection of the canal space relies on irrigation materials and methods, as well as on intracanal medicaments. Previous studies have shown that calcium hydroxide can reduce dentin resistance to fractures, to which immature permanent teeth are more prone [32]. This supports the need for expanding the options of intracanal medicaments to be used in REPs that can replace calcium hydroxide as the gold standard. Another potential disadvantage of calcium hydroxide relates to the inactivation of its antimicrobial activity, especially against *E. faecalis*, by dentin products [33]. The antimicrobial activity of HICA was evaluated against *E. faecalis* in the presence of dentin powder and was shown to be retained against *E. faecalis* in both nutrient-deficient and nutrient-rich environments. HICA was also shown to resist inactivation by dentin in a dose-dependent manner [25]. Other commonly used intracanal medicaments, namely triple antibiotic paste (TAP) and modified triple antibiotic paste (mTAP), have been shown to have antimicrobial efficacy against *E. faecalis* [13]. However, their potential side effects and damage to the resident stem cells remain of concern. A recent systematic review evaluating the in vitro cytotoxicity of TAP, DAP, and calcium hydroxide on SCAP concluded that the use of antibiotic pastes lead to a reduction in SCAP survival compared to calcium hydroxide, although distinctions could not be made between the antibiotic pastes [20]. Other potential complications of antibiotic pastes are the development of resistant organisms, interference with the release of dentinal growth factors, inhibition of vascularization, and high toxicity levels [12]. The search for alternative natural intracanal medicaments is ongoing, and one such candidate that has been studied more extensively is propolis. A study evaluating the antibacterial activity of TAP, mTAP, calcium hydroxide, and ethanol extract of propolis (EEP) found that EEP and calcium hydroxide are equally effective antimicrobials as TAP and mTAP [16]. However, given the potential allergenic properties of propolis, additional naturally occurring compounds, such as HICA, should be further explored for usage in REPs.

In addition to its better antimicrobial properties than calcium hydroxide against *E. faecalis*, HICA has been shown to be effective against most Gram-positive and Gram-negative species [24,25,28]. A study by Fouad evaluated the organisms associated with immature traumatized infected teeth, which are candidates for REPs, and found that a high proportion of strictly anaerobic bacteria are present [34]. *Fusobacterium nucleatum* was found to be present in 30% of cases evaluated and HICA has been shown to be bactericidal against *Fusobacterium nucleatum* [28]. However, additional clinical studies supporting the use of HICA as an intracanal medicament in REPs are needed.

The biggest challenge in REPs is the need to perform effective disinfection while maintaining a suitable microenvironment in REPs for SCAP to proliferate and differentiate. This in vitro study evaluated the effects of HICA, as a potentially novel intracanal medicament, on the survival and differentiation of SCAP. Since intracanal medicaments used in REPs must have maximum antimicrobial efficacy without being detrimental to the stem cells, the first part of our study evaluated the direct exposure of SCAP to varying concentrations of HICA and compared it to calcium hydroxide as the gold standard. HICA at concentrations of 1–10 mg/mL and Ca(OH)_2_ at 1 mg/mL elicited no significant cell death compared to the no treatment control, regardless of the length of exposure time. This suggests that the concentration of intracanal medicament is more important to SCAP survival than the duration of contact [8]. However, the SCAP viability decreased gradually in a dose-dependent manner in the HICA-treated groups. Based on these results, HICA concentrations above 1 mg/mL could be cytostatic but not cytotoxic to SCAP. 

The DNA damage to SCAP was assessed via the detection of two immunofluorescent markers for DNA double-strand breaks (DSBs), γH2AX and 53BP1. Genotoxic substances compromise the integrity of DNA by inducing DSB, which unrepaired are a source of genomic instability [35]. The chromatin surrounding these DBS undergoes extensive phosphorylation, specifically on a serine residue on histone H2AX, which in turn recruits repair proteins, such as 53BP1, at the site of the damage to initiate the repair process [35,36]. Additionally, γH2AX cannot be used as sole marker for DBS, as it is detected in cellular senescence or arrest of the replication fork when DBS are not present [37]. Therefore, we used the detection of co-localized protein 53BP1 to increase the sensitivity of the DBS quantification. HICA at concentrations above 5 mg/mL induced significant DNA damage compared to the control, similar to the findings of the study by Selis et al. on human periodontal ligament fibroblasts [30]. The dose-dependent introduction of DSB could result in cell cycle arrest while DNA damage is repaired and could explain the cytostatic but non-cytotoxic effect of low HICA concentrations. SCAP viability is critical for the secondary objective of continued root development and maturation [38]. Therefore, the finding of this study that SCAP viability can be maintained is critical in helping to preserve the microenvironment of the apical area of the canal space to allow the continued regeneration and maturation of the root [39].

The second part of our study evaluated the differentiation potential of SCAP in the presence of HICA. The activity of early and late mineralization markers—namely ALP and Alizarin Red, respectively—were reduced at 1 mg/mL of HICA by about 30–60% compared to untreated cells. Surprisingly, the expression of markers for various stages of osteogenic and odontogenic differentiation (*RUNX2*, *OCN*, *BSP1*, and *DSPP1*) appeared to have specific expression patterns in SCAP exposed to 1 mg/mL of HICA in differentiation media; *RUNX2* expression increased, *OCN* decreased, while *BSP1* and *DSPP1* remained at levels similar to those in induction media without HICA. Each one of these markers has a very specific temporal pattern of expression, and although ALP and Alizarin Red were evaluated at the peak of their activity, the gene expression was determined at 7 days, even though their temporal expression levels vary during the differentiation process [40,41,42]. Since protein translation in osteoblasts seems to be pH-dependent, with a lower pH decreasing translation but not transcription [43], we proposed that since HICA is highly acidic, the mRNAs are produced but not converted into active protein. Additionally, a similar pattern of increased *BSP1* expression without increased Alizarin Red staining was observed in SCAP exposed to LPS, suggesting the importance of the cellular environment in differentiation [44]. This discrepancy in osteogenic gene expression and ALP and Alizarin Red activity was previously observed in dental pulp stem cells exposed to melatonin and could warrant further examination [45]. Since increased mineralization was achieved in SCAP treated with 1 mg/mL of HICA, further studies are necessary to understand the effect of HICA on differentiation by determining the mRNA and protein levels of all tested markers when exposed to 1 mg/mL of HICA for 7, 14, and 30 days.

## 5. Conclusions

Within the limitations of this in vitro study, we have shown that HICA at 1 mg/mL of HICA is equally cytotoxic and genotoxic to calcium hydroxide and does still allow for the retention of most of the odontogenic and osteogenic potential of SCAP. Therefore, HICA could be an alternative to calcium hydroxide as an intracanal medicament for REPs.

## Figures and Tables

**Table 1:** Primer sequences for qRT-PCR.

Gene	Forward Primer	Reverse Primer
*DSPP1*	GGGACACAGGAAAAGCAGAA	TGCTCCATTCCCACTAGGAC
*BSP1*	ATGGAGAGGACGCCACGCCT	GGTGCCCTTGCCCTGCCTTC
*OCN*	GACTGTGACGAGTTGGCTGA	AAGAGGAAAGAAGGGTGCCT
*RUNX2*	CCCGTGGCCTTCAAGGT	CGTTACCCGCCATGACAGTA
*GAPDH*	GAAGGTGAAGGTCGGAGT	GAAGATGGTGATGGGATTTC

## Data Availability

Data unavailable due to privacy.
